# Powering Up the Brain: Intercellular Mitochondrial Quality Control and Its Implication in Stroke

**DOI:** 10.2174/011570159X388351250620065716

**Published:** 2025-06-30

**Authors:** Xinyu Zhou, Shenzhe Wu, Tianxi Huang, Yue Li, Jianhong Yang, Zhen Gu, Xiangnan Zhang

**Affiliations:** 1 State Key Laboratory of Advanced Drug Delivery and Release Systems, College of Pharmaceutical Sciences, Institute of Pharmacology & Toxicology, Zhejiang University, Hangzhou, 310058, China;; 2 State Key Laboratory of Advanced Drug Delivery and Release Systems, Institute of Pharmaceutics, College of Pharmaceutical Sciences, Zhejiang University, Hangzhou, 310058, China;; 3 Jinhua Institute of Zhejiang University, Jinhua, 321299, China;; 4 Department of Neurology, The First Affiliated Hospital of Ningbo University, Ningbo, 315010, China;; 5 Department of Chemical Biology, Ernest Mario School of Pharmacy, The State University of New Jersey, Rutgers, Piscataway, NJ, 08854, USA

**Keywords:** Intercellular mitochondrial quality control, neuronal survival, autophagy, intercellular mitochondrial transport, ischemic conditions, trans mitophagy, stroke

## Abstract

Mitochondria are critical for neuronal survival and function, and their dysregulation is closely related to the incidence and prevalence of various neurological disorders, including stroke. Mitochondrial quality control (MQC) is vital for maintaining mitochondrial integrity, particularly in neurons. Under ischemic conditions, neurons evolve a range of adaptive strategies to preserve mitochondria function dynamically, either by generating functional mitochondria or by eliminating dysfunctional ones *via* autophagy, both of which play key roles in keeping neuronal survival under the conditions of stroke. Besides these intracellular strategies, the intercellular mechanisms underlying MQC have been observed in the nervous system. Functional mitochondria from healthy cells can be supplemented to ischemic neurons in distinct manners and thus restore the mitochondrial network of recipient cells. Conversely, injured neurons release dysfunctional mitochondria, which can be further degraded by adjacent glial cells. Alternatively, the discarded mitochondria act as a threat to surrounding cells and can disrupt the homeostasis of the nervous system. In this review, the key discoveries in intercellular MQC in the nervous system were summarized, and further discussed the implications of intercellular MQC strategies for stroke therapy.

## 1 INTRODUCTION

Mitochondria are essential to neurons not only for their bioenergetic functions but also for roles in calcium buffering, metabolism, and regulation of cell fate. Consequently, mitochondrial quality control (MQC) is crucial for maintaining the nervous system homeostasis, and mitochondrial dysfunction is closely associated with a variety of neurological disorders, including Parkinson's disease (PD), Alzheimer's disease (AD), amyotrophic lateral sclerosis (ALS), and stroke [1-4]. Neurons, as long-lived cells with lifespans equivalent to that of the individual, cannot eliminate defective mitochondria through cell division. Therefore, they have developed several strategies to preserve the mitochondrial homeostasis. Besides mitochondrial biogenesis, neurons remove dysfunctional mitochondria *via* the autophagosome-lysosome pathway, a process referred to as mitophagy [5]. The link between mitophagy defects and neurological disorders was first established through extensive studies on the mitophagy-related protein Parkin. Loss-of-function mutations in Parkin lead to the accumulation of dysfunctional mitochondria in dopaminergic neurons, resulting in familial onset of PD [6]. Furthermore, proteins involved in mitophagy, such as Ambra1, Fundc1, Bnip3, and Bnip3L/Nix, have been implicated in several neurological disorders, including neural tube defects, diabetic cognitive impairments, and stroke [7-9]. Although the precise molecular mechanisms underlying neuronal mitophagy remain incompletely elucidated, accumulating evidence suggests that insufficient mitophagy leads to neuronal dysfunction.

In addition to the intracellular MQC mechanisms outlined above, emerging studies indicate that mitochondria can be transported and/or degraded between cells within the nervous system. Functional mitochondria can be transferred from healthy cells to stressed neurons through distinct mechanisms, thereby restoring the mitochondrial network in recipient cells [10-12]. In contrast, dysfunctional mitochondria can be actively removed from injured neurons and subsequently degraded by surrounding astrocytes [13]. A common feature of these MQC activities is the intercellular transport of mitochondria, which supports the biological function of the mitochondrial network. These intercellular MQC strategies seem to play significant roles in preserving nervous system homeostasis.

This review aims to summarize the processes and regulatory mechanisms underlying intercellular MQC in the nervous system and discuss the implications of this phenomenon for the clinical therapy of related diseases. In this narrative review, the PubMed database was used to search peer-reviewed publications for summarizing the functional roles of intercellular mitochondrial quality control in stroke using the following terms: ‘stroke’, ‘mitophagy’, ‘transmitophagy’, ‘mitochondrial quality control’, ‘mitochondrial transport’, ‘tunneling nanotubes’, and ‘extracellular vesicles’ in combination with mitochondrial motor proteins. This study also performed a bibliographic search for the clinical impact of the therapeutic strategies targeting mitochondrial transfer, using the keywords such as ‘mitochondria transplantation’ and ‘mitochondria isolation’ in combination with ‘ischemia’, ‘treatment’, ‘therapy’, and equivalent terms. In addition, reference lists of primary and review articles were reviewed for additional relevant publications, while excluding non-English articles, conference abstracts, and studies unrelated to the topic. All relevant publications until January 2025 were critically evaluated and discussed on the basis of relevance, quality, and timelines.

## 2 MITOCHONDRIAL MOBILITY AND QUALITY CONTROL

### 2.1 Intracellular Mitochondrial Transport

 Neurons, the basic functional units of the central nervous system, exhibit a distinct cellular structure comprising a soma, an axon, and dendrites. A notable morphological feature of neurons is the unique distribution of mitochondria, which are not restricted to the soma but are also extensively distributed throughout axons and dendrites. This distribution is particularly pronounced in regions with high ATP demands, such as presynaptic and postsynaptic terminals, active growth cones, axonal branches, and nodes of Ranvier [14]. The localization of mitochondria is crucial to meet the energy supply and metabolic needs of neurons.

Effective mitochondrial transport within axons and dendrites is indispensable for nerve signal conduction and synaptic function. Beyond serving as cellular energy factories, mitochondria are involved in regulating intracellular calcium levels, maintaining redox balance, modulating apoptosis, and various physiological processes. In synapses, ATP provided by mitochondria supports neurotransmission, thereby sustaining normal synaptic function. Furthermore, the transport and distribution of mitochondria are intricately linked to neuronal morphological development and plasticity [15]. Mitochondrial transport within the growth cone facilitates axonal growth and orientation.

**Fig. (1):**
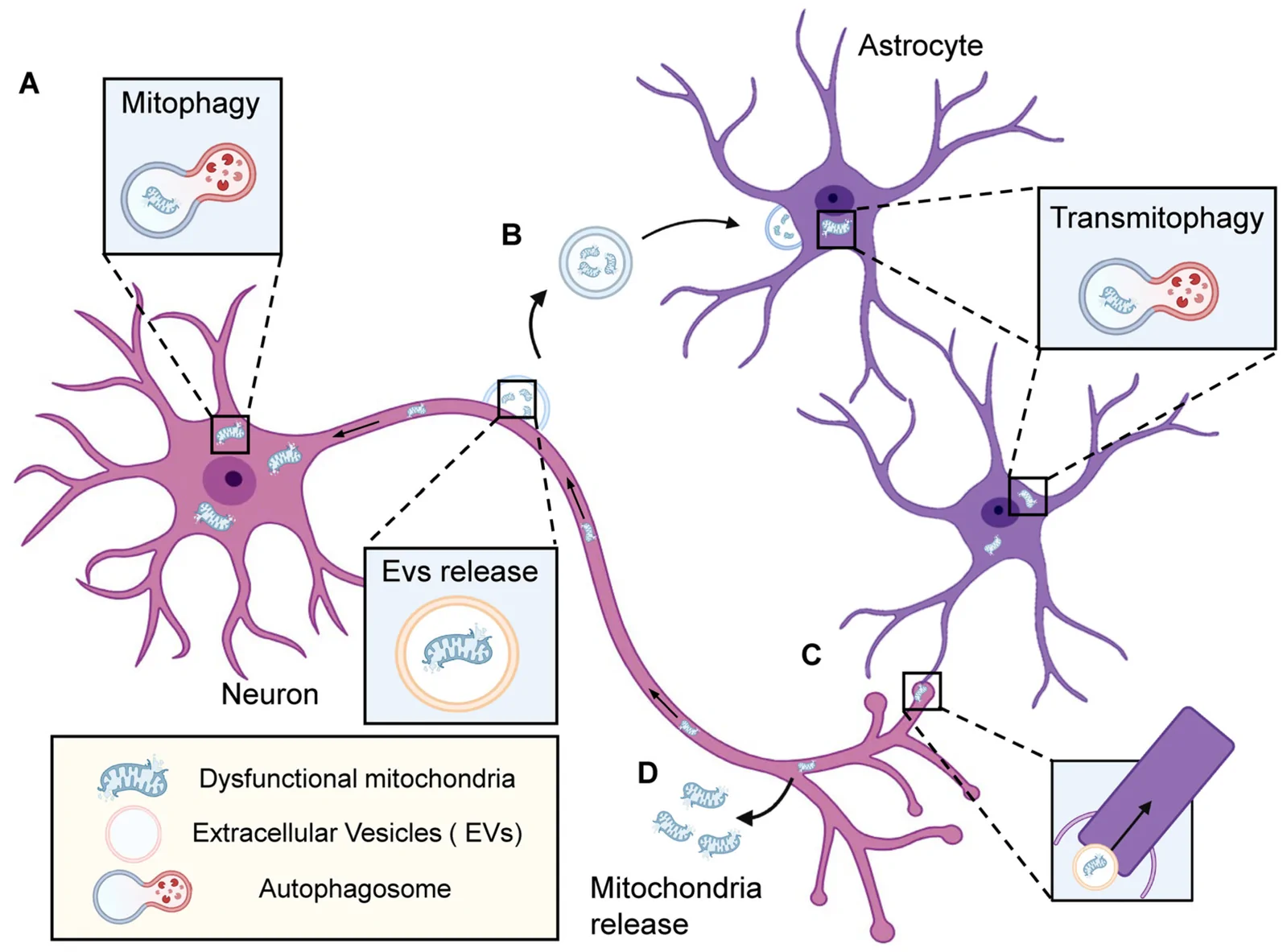
An overview of mitochondrial transfers in ischemic neurons. Dysfunctional mitochondria in neurons are engulfed and degraded by phagocytes. (**A**) Axonal mitochondria undergo retrograde transport and are recognized by the autophagosome in the neuronal soma. (**B**) Mitochondria within the autophagosome accumulate in protrusions at neuron terminals and are then digested through mitophagy in astrocytes. (**C**) Mitochondria within the autophagosome accumulate near astrocytes and are subsequently released in vesicles. Once internalized by astrocytes, extracellular vesicles (EVs) fuse with lysosomes, where the contents are digested. (**D**) Injured mitochondria are released into the extracellular milieu. The figure is created using biorender.

In pathological conditions, particularly in ischemic brains, mitochondrial transport undergoes significant alterations [16]. Ischemia induces an energy crisis in neurons, disrupting mitochondrial transport mechanisms. Under ischemic conditions, mitochondrial transport in axons may be impaired, leading to the accumulation of mitochondria in specific regions of the soma or axon [17]. This transport disruption further contributes to mitochondrial dysfunction by impeding the proper distribution and function of mitochondria within neurons, resulting in impaired ATP production, calcium dysregulation, and heightened oxidative stress [18-20]. Additionally, during ischemia-induced autophagy, mitochondrial transport interacts with autophagosome formation and movement, influencing the clearance of damaged mitochondria [21]. Specifically, disrupted mitochondrial transport under ischemic conditions can impair the delivery of damaged mitochondria to autophagosomes, thereby reducing the efficiency of autophagic clearance [22, 23]. Conversely, the formation and movement of autophagosomes can alter the cytoskeletal dynamics and cellular environment, which in turn affects mitochondrial transport and distribution [24, 25]. Alterations in mitochondrial transport are also critical in neurodegenerative diseases. In AD and PD, disruptions in mitochondrial transport led to abnormal mitochondrial distribution within neurons, impairing nerve signal conduction and synaptic function [26, 27]. These transport disruptions are closely associated with neuronal apoptosis and neural network degeneration (Fig. **1A**).

### 2.2 Intercellular Mitochondrial Transport

 Recently, studies have described a process termed transmitophagy, wherein dysfunctional mitochondria are degraded by adjacent glial cells, particularly astrocytes [13]. In healthy retinal ganglion cells, damaged mitochondria accumulate in axonal protrusions, which are subsequently shed along with other axoplasmic components and membranes. These vesicles are later endocytosed and degraded by astrocytes [28, 29] (Fig. **1B**). Similar mitochondrial clusters have been observed in the superficial layers of the cerebral cortex, implying that transmitophagy may occur in these regions as well [13]. In dopaminergic neurons (DANs), defective mitochondria are loaded into axonal fragments, which are subsequently taken up by astrocytes that infiltrate the neurons, ultimately leading to degradation in lysosomes [30]. However, in Alzheimer's disease, this process is significantly altered. In AD mouse models, the internalization and degradation of neuronal mitochondria by astrocytes are enhanced from the age of six months onward, potentially preventing injured mitochondria from inducing neuroinflammation [31].

 Transmitophagy involves the accumulation of dysfunctional mitochondria in neuronal protrusions, followed by detachment of these protrusions or their penetration by astrocytes and, ultimately, the transfer and elimination of mitochondria within astrocytes [29]. The accumulation of dysfunctional mitochondria in neuronal protrusions has been observed in both physiological and pathological conditions, with an increased removal of these damaged mitochondria *via* transmitophagy under pathological conditions. This implies that defective mitochondrial transport and subsequent accumulation of dysfunctional mitochondria may contribute to transmitophagy [32]. The detachment of protrusions involves several mechanisms, including mito-lysosome exocytosis, migrasome-mediated mitocytosis, and mitoptotic body-dependent elimination [33, 34]. Mito-lysosome exocytosis involves the formation of lysosome-like vesicles that engulf damaged mitochondria and release their contents into the extracellular space [35, 36]. Migrasome-mediated mitocytosis involves the capture and transport of mitochondria by migrasomes, which are vesicles that form at the tips of cellular protrusions during cell migration [37]. Finally, mitoptotic body-dependent elimination involves the formation of specialized structures around damaged mitochondria, which are then recognized and engulfed by neighboring cells for degradation [38] (Fig. **1C**). Despite the significant reduction of mitolysosome due to treatment with the lysosome inhibitor chloroquine (CQ), transmitophagy still occurred [34]. Additionally, the uptake of mitochondria-enriched vesicles from retinal ganglion cells differs from that of fragments from DANs, likely due to the size discrepancies between the vesicles. Spheroids derived from DANs are significantly larger and more difficult to internalize than those from retinal ganglion cells [13, 30].

Under physiological conditions, most mitochondria in retinal ganglion cells are removed *via* trans mitophagy rather than mitophagy, which is generally assumed to degrade the majority of mitochondria [13]. This process is enhanced under stress and has only been observed in the nervous system, specifically in unmyelinated neurons, such as retinal ganglion cells, DANs, and touch receptor neurons. Cells with high phagocytic activity, such as astrocytes and microglia, are capable of acting as recipient cells [13, 30, 39]. These findings suggest that trans mitophagy is a highly specialized biological process, possibly adapted by the nervous system to meet high energy demands and compensate for the limitations of axonal mitochondrial transport in conventional neuronal mitophagy. Retinal ganglion cells, in particular, are energy-demanding and require a level of mitochondrial turnover that exceeds axonal transport capacity, distinguishing them from other neurons that primarily rely on this pathway for mitochondrial degradation [40]. This phenomenon raises several intriguing questions: Is the transcellular degradation process exclusive to mitochondria? How do mitochondria assemble at appropriate axonal locations and load themselves into vesicles for degradation? What mechanisms allow astrocytes to distinguish between axon vesicles containing impaired mitochondria and healthy axon substances, ensuring correct uptake and degradation? These questions remain open for further investigation.

 While trans mitophagy contributes to maintaining nervous system homeostasis by eliminating dysfunctional mitochondria, injured mitochondria and their components can also be released into the extracellular milieu in acquired brain injury and neurogenerative diseases, acting as damage-associated molecular patterns (DAMPs) and disrupting normal cell activity [41] (Fig. **1D**). This may result from deficiencies in the elimination of impaired mitochondria [42], leading to propagation of neuroinflammation [43]. Excessive mitochondrial fission within microglia, mediated by the Drp1-Fis1 interaction, can impair microglial function and activate them into an inflammatory phenotype. These activated microglia release injured mitochondria and/or their fragments into the extracellular milieu, inducing pathological mitochondrial fragmentation and dysfunction in astrocytes [44]. Consequently, naïve astrocytes are activated to the A1 state, a pro-inflammatory phenotype characterized by the upregulation of pro-inflammatory molecules and the loss of neuroprotective functions, rapidly inducing neuronal injury and cell death [45]. The uncontrolled glia activation driven by the release of mitochondrial debris amplifies the severity of brain injury, while DAMPs from dead neurons further exacerbate the situation. In another scenario, mitochondria have been shown to bind to and be internalized by platelets, disrupting their normal activity. During this process, cardiolipin (CL), a lipid that promotes coagulation, translates from the mitochondrial inner membrane to the surface of damaged mitochondria. This translocation of CL can disrupt the endothelial barrier, causing vascular leakage and compromising the integrity of the blood-brain barrier, thereby exacerbating neurological conditions [46]. Additionally, mitochondria-derived reactive oxygen species (ROS) can also activate platelets, promoting coagulation [47]. As a result, fibrinogen, essential for clotting, is depleted, impairing blood clotting and contributing to coagulopathy.

 It is noteworthy that while damaged mitochondria in the extracellular milieu are detrimental, the transfer of functional mitochondria offers protective benefits. The total quantity of discharged mitochondria has been reported to remain stable in several cases [45]. The differing outcomes of these processes may be determined by the ratio of functional to dysfunctional mitochondria among those released mitochondria. When a higher proportion of defective mitochondria are released than functional ones, the process may become neurotoxic. In conclusion, strategies that reduce the proportion of dysfunctional mitochondria and increase the functional ones among all released mitochondria may have neuroprotective effects in related neurological disorders [45]. The clearance of vesicles containing damaged mitochondria could also help mitigate the situation (Fig. **1**) [46].

## 3 THE INTERCELLULAR SUPPLEMENT OF FUNCTIONAL MITOCHONDRIA IN CEREBRAL ISCHEMIA

**Fig. (2):**
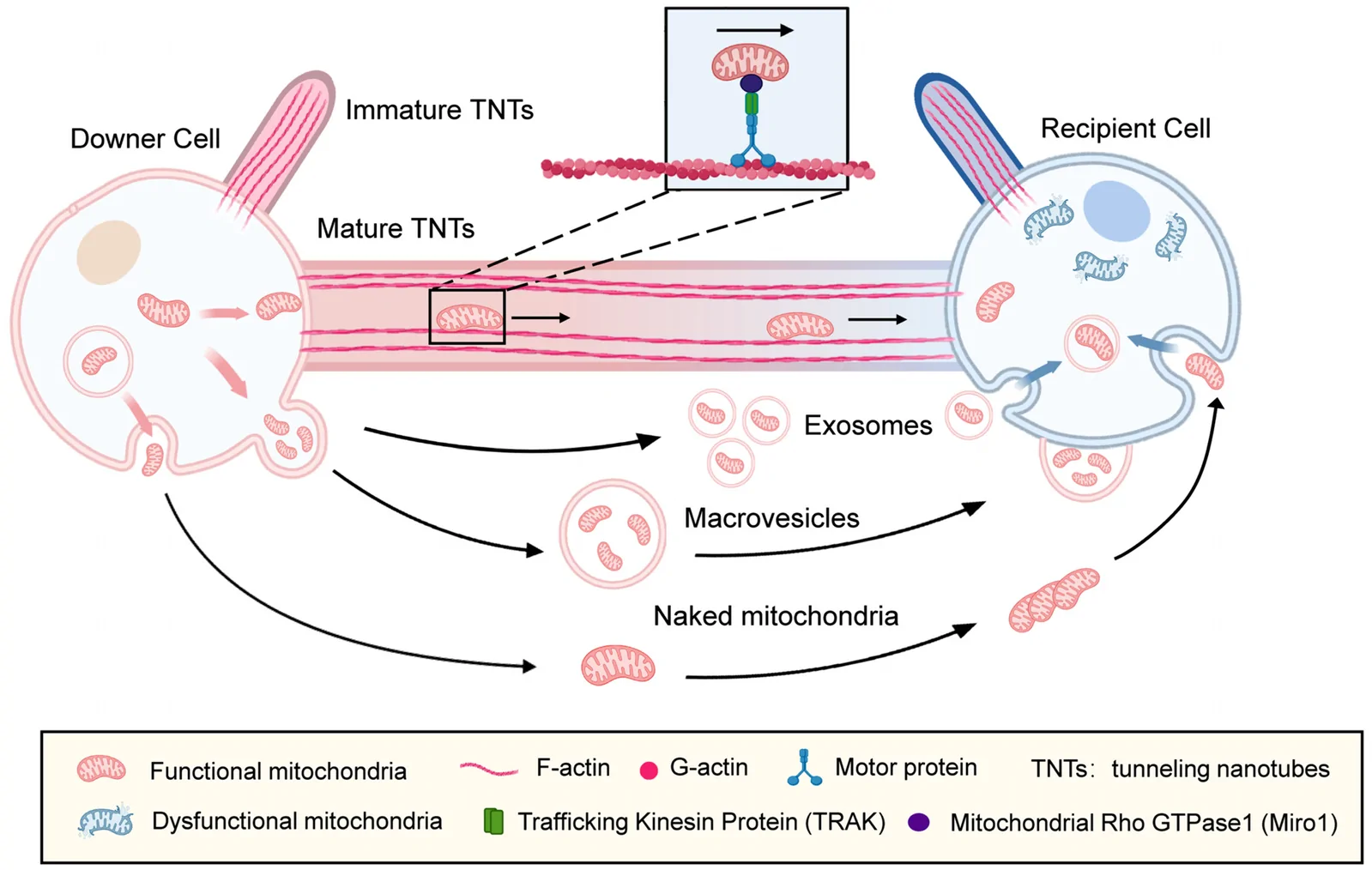
Intercellular supplement of functional mitochondria. Intracellular mitochondrial transfer is primarily mediated through three mechanisms: tunneling nanotubes (TNTs), extracellular vesicles (EVs), and direct transfer of naked mitochondria. TNTs are intercellular membrane tubules composed of F-actin, facilitating the directed transfer of mitochondria *via* the microtubule transport system. Mitochondrial transport appears to depend on cytoskeleton components such as F-actin, as well as the Miro-1 and its interaction with the trafficking kinesin protein (TRAK) and motor protein. EVs, a heterogeneous class of vesicles that includes exosomes and microvesicles, can carry intact mitochondria or mitochondrion-derived vesicles. They promote the repair of recipient cells by transferring mitochondria between cells through secretion and uptake. In contrast, naked mitochondrial transfer involves the direct release of mitochondria from donor cells into the extracellular environment, followed by uptake by recipient cells. These transfer modes play crucial roles in intercellular material exchange and signal transmission and are closely associated with the pathogenesis and progression of various diseases. → indicates the direction of moving. The figures are created using biorender.

 The initial observation of intercellular functional mitochondrial transport was documented in a cardiomyogenic differentiation model. In this model, living mitochondria were observed moving from cardiomyocytes to endothelial progenitor cells *via* intercellular nanotubular connections, enabling the endothelial progenitor cells to acquire a cardiomyogenic phenotype [48]. Beyond their role in mediating differentiation, exogenous mitochondria have the potential to rescue recipient cells by integrating and collaborating with the endogenous injured mitochondrial network [49]. Intercellular mitochondrial transport within the nervous system has emerged as a prominent area of research due to its significant therapeutic potential. This process is characterized by complex mechanisms that facilitate mitochondrial transfer between cells through various means, including the formation of tunneling nanotubes (TNTs), the release of extracellular vesicles (EVs), and other potential mechanisms (Fig. **2**). The transfer of mitochondria is particularly crucial for restoring the metabolic function and proliferative capacity in cells damaged by cerebral ischemia. The efficiency and direction of this mitochondrial transfer are tightly regulated by various intracellular signaling pathways and oxidative stress conditions. Consequently, mitochondrial transfer not only plays a central role in the pathophysiological mechanisms underlying cerebral ischemia but also represents a promising therapeutic target for the development of new treatments. This approach holds the potential for alleviating neurological dysfunction associated with cerebral ischemia (Table **1**).

### 3.1 Intercellular Mitochondria Transport via Tunneling Nanotubes (TNTs)

 Tunneling nanotubes (TNTs) refer to intercellular communication channels that directly connect the cytoplasm of adjacent cells, facilitating the exchange of cellular contents and enabling complex cellular interactions. Structurally, TNTs are composed of internal cytoskeletal elements, such as F-actin or microtubules, along with external membrane structures [50]. These structures are crucial for the exchange and communication of substances, including mitochondria, between various cell types, particularly within the central nervous system [51-53].

 The initial discovery of functional TNTs *in vitro* in rat PC12 cells led to subsequent studies identifying their presence across diverse cell lines in CNS, including primary neurons, astrocytes, pericytes, and microglia [54-56]. This extensive distribution highlights the crucial role of TNTs in diverse cellular environments. TNTs are highly responsive to external stimuli, with their formation significantly increasing under conditions of oxidative stress, inflammation, and other adverse circumstances [57-59]. This intercellular connectivity serves as a vital adaptive mechanism, allowing cells to share resources and enhance survival under stress.

 In stroke models, the transfer of functional mitochondria *via* TNTs was first observed between multipotent mesenchymal stem cells and neuron-like PC12 cells, which improved the viability of recipient cells and mitigated the ischemic damage [60]. With the advancement of 2D cell co-culture and 3D neutrosphere culture techniques, extensive crosstalk within the neurovascular unit *via* TNTs was discovered, wherein functional mitochondria are transferred to protect against ischemic injury. Neurons, particularly vulnerable to ischemia-reperfusion injury, are further compromised by subsequent pathological processes such as inflammation, excitotoxicity, and oxidative stress. Under ischemic stress, healthy neural stem cells, astrocytes, microglia, and pericytes extend TNTs to deliver functional mitochondria to compromised neurons, rescuing them from cell death and restoring their biological functions [61-64]. For instance, astrocytes have been shown to transfer healthy mitochondria to injured neurons *via* TNTs, aiding recovery from cobalt nanoparticles (Co-NPs) -induced neurotoxicity [65]. Similarly, photoreceptors can provide functional mitochondria through TNTs, thereby restoring vision function [11].

Under stress conditions, an increased transfer of functional mitochondria *via* TNTs has been reported, while the reverse transport of dysfunctional mitochondria is suppressed [66]. Emerging studies suggest that increased intercellular mitochondria transport is associated with the formation of more TNTs [62]. The formation of TNTs involves several steps, including biosynthesis, recognition of immature TNTs from donor to recipient cells, and the maturation of these structures into functional channels. Ischemic endothelial cells have been observed to form more immature TNTs when co-cultured with mitochondria donor cells compared to cells under normal conditions [66]. A similar phenomenon has been reported between neural cells and multipotent mesenchymal stem cells [60]. Although the molecular regulation of immature TNT recognition and connection remains elusive, certain receptor-ligand interactions have been implicated. For instance, increased surface-exposed phosphatidylserine on injured cells promotes the maturation of immature TNTs [66]. 

Additionally, S100A4, a hypoxia-induced calcium-binding protein, can direct TNT formation by interacting with its receptor and determining target cells [67]. Studies have shown that endoplasmic reticulum protein 29 (ERp29) acts as a molecular chaperone that promotes the formation of TNTs by stabilizing the TNF-α-induced protein 2 (TNFAIP2) [68]. However, the mechanisms underlying intercellular mitochondria transport between neurons and their donor cells require further investigation. Bidirectional mitochondria movement under physiological conditions can shift to unidirectional traffic from healthy to impaired cells under pathological conditions [66]. It is suspected that mitochondria transport from damaged to intact cells is disrupted under stress, potentially due to the clearance of Miro1, a protein essential for mitochondria movement, from injured cells [32].

When intercellular mitochondria transport *via* TNTs is obstructed, increased neuronal apoptosis and disease progression may occur [65]. Deficiencies or loss-of-function mutations in molecules essential for TNT biogenesis, stability, and cargo transport, such as CD38, Wnt ligands, Eps8, Arp2/3 activators, and Miro1, have been implicated in neurological disorders, potentially contributing to these diseases by impairing intercellular mitochondria transport [69-73]. Cell transplantation has been explored as a therapy for neurological disorders like stroke and optic neuropathy, with its effectiveness partially attributed to the intercellular supplementation of functional mitochondria *via* TNTs [11, 60]. Enhancing this process by upregulating the expression or activity of essential proteins, such as Miro1, in TNTs-dependent mitochondria transfer has shown therapeutic potential [60]. However, recent studies have highlighted potential risks associated with enhanced intercellular mitochondria transport, as the exchange of viruses and prion aggregates *via* TNTs may facilitate the spread of pathogens between cell's nanotubular connections [74, 75]. This poses unexpected risks in neurodegenerations related to prions [76].

### 3.2 Intercellular Mitochondria Transport via Extracellular Vesicles (EVs)

 Extracellular Vesicles (EVs) encompass a variety of membrane-bound phospholipid vesicles released by cells, including micro-vesicles (MVs) (ranging from 100 nm to 1 μm), exosomes (ranging from 30 nm to 100 nm), and apoptotic bodies (ranging from 1 μm to 2 μm). Initially, it was believed that only micro-vesicles were large enough to transport organelles like mitochondria. However, recent studies have demonstrated that mitochondria can co-localize with protein markers not only in micro-vesicles but also in exosomes within the nervous system. This co-localization is considered crucial for communication during various pathophysiological events [77].

The first evidence suggesting that EVs may carry mitochondrial components emerged in 2010 when mitochondrial DNA (mtDNA) was identified in EVs derived from cultured astrocytes and glioblastoma cells [78]. Subsequent research confirmed the presence of various mitochondrial components in EVs, including mtDNA fragments, full-length mtDNA, mitochondrial proteins, and even intact mitochondria, across different cell types [79]. Isolated mitochondria-enriched EVs from brain endothelial cells were characterized by distinct physicochemical properties, membrane integrity, and protein composition. These findings revealed that intact mitochondria were present only in medium-to-large EVs (m/lEVs), which are defined as micro-vesicles with a diameter greater than 200 nm, whereas small EVs (sEVs) with smaller diameter contained only mitochondrial proteins without intact mitochondria [79]. Although the mechanisms by which mitochondrial components are loaded into EVs are still unknown, selective packaging of mitochondria may occur during EV formation [41].

Mitochondrial transport *via* EVs has been observed in a variety of pathological conditions. Energy consumption is vital for maintaining the homeostasis of central nervous system functions. In the context of cerebral ischemia, neuronal mitochondrial impairment leads to significant energy deficits, as damaged mitochondria produce excessive ROS and release pro-inflammatory factors, exacerbating the energy crisis and contributing to further brain damage. In this case, the appropriate source of EVs can effectively mitigate the neuronal injury caused by ischemia. Several studies have documented the transfer of functional mitochondria after stroke, highlighting their therapeutic benefits in promoting neurogenesis, rescuing aerobic respiration, reducing oxidative stress, elevating ATP levels, and facilitating angiogenesis [80-82]. For instance, healthy mitochondria can be transported *via* micro-vesicles and exosomes from astrocytes to ischemic neurons, enhancing neuronal survival and plasticity [83].

Additionally, mitochondria can be transferred from neural stem cells to mtDNA-deficient L929 Rho0 cells and pro-inflammatory mononuclear phagocytes *via* exosomes, recovering normal mitochondrial activity in recipient cells [10]. The process of EV-dependent intercellular mitochondria transport involves several steps, from cargo loading and EV biogenesis to internalization by recipient cells. Miro1 and Miro2 are thought to recruit mitochondria to vesicle formation sites [84]. CD63 plays a critical role in cargo loading within exosomes, as mitochondria from neural stem cells cannot be loaded into CD63-depleted EVs [10, 85]. The CD38/cADPR/Ca^2+^ pathway regulates EV release in astrocytes and neurons, with CD38 levels reported to increase after stroke, enhancing intercellular mitochondria transport and providing neuroprotection [83, 84]. The chaperone protein CCT can influence the biogenesis of EV by regulating the dynamics of the driver protein, which in turn affects the lipid composition and metabolic pathway of cells. Some molecular chaperones, including HSP70 and HSP90, have been found to be packaged into EVs and passed between cells [86, 87]. For EV internalization, dynamin- and clathrin-mediated endocytosis, as well as integrin-Src/Syk signaling-mediated endocytosis, have been described in the nervous system [10, 83]. Impairments in these biological processes can reduce the quantity of intercellularly transported mitochondria. Suppression of CD38 signaling and mutations that impair CD38 expression has been demonstrated to disrupt this process and worsen neurological outcomes [83, 84, 88]. Deficiencies in Miro1 or Miro2 reduce the efficiency of mitochondrial transfer *via* EVs [84], and endocytosis inhibitors can also prevent exogenous mitochondria from entering recipient cells [10, 12, 83].

 Similar to the intercellular transport of functional mitochondria *via* TNTs, the EV-dependent process is also selective, with EVs prone to be released by certain cell types and internalized by others. The majority of mitochondria-loaded EVs from neural stem cells are internalized by mononuclear phagocytes and astrocytes, while neurons, oligodendrocytes, and T-cells receive only a minor fraction of EVs in co-culture models [10]. Although bidirectional mitochondrial movement has been observed between astrocytes and neurons, mitochondrial transfer efficiency is much higher when EVs move from astrocytes to neurons compared to the reverse [84]. These differences are thought to arise from variations in cell metabolism and phagocytosis. Specifically, neural stem cells and astrocytes, which are highly glycolytic, are more likely to provide mitochondria than cells that rely on mitochondrial respiration, such as neurons [89-91]. Recipient cells with phagocytotic functions are more active in incorporating mitochondria-loaded EVs [92]. However, EVs may not be the sole mechanism for mitochondria transport in cells with these characteristics, as mesenchymal stem cells, which are glycolytic-dependent, transfer mitochondria *via* TNTs [66]. The factors influencing cells to choose specific pathways for intercellular mitochondria transport are still obscured.

 Therapies based on intercellular mitochondria transport *via* EVs are progressively being developed. Neural stem cell transplantation has been shown to contribute to recovery from multiple sclerosis, at least partially, by producing functional mitochondria-loaded EVs [10]. Injection of purified EVs has also been shown to restore heart function after myocardial infarction, suggesting potential therapeutic applications for cerebral ischemia [93]. In addition, activators of EV-related signaling pathways are considered promising for treating neurological disorders. Glutamate and protein kinases 1/2 have been reported to activate CD38 expression, thereby exerting therapeutic effects [88, 94].

### 3.3 Intercellular Naked Mitochondria Transport

Besides being transported through TNTs and EVs, mitochondria can also be released into the extracellular milieu nakedly and then be internalized by adjacent recipient cells [10, 65]. Naked functional mitochondria have been shown to transfer from endothelial progenitor cells to brain endothelial cells *via* conditioned medium, thereby alleviating cellular impairment *in vitro* [95]. Furthermore, the direct injection of functional mitochondria into disease-affected brain regions or veins can be internalized by neurons. These strategies are capable of restoring mitochondrial function and offer potential therapeutic benefits for neurological disorders, such as stroke, PD, and traumatic brain injury (TBI) [96-98]. However, it remains unclear whether naked mitochondria transport can occur spontaneously *in vivo*, particularly within the nervous system.

 The mechanisms governing the release, internalization, and survival of mitochondria during intercellular transport have been partially elucidated. In platelets, mitochondrial release is associated with actin, which serves as a track for organelle movement [99, 100]. Different cell types employ various endocytic pathways for the internalization of naked mitochondria. For instance, HepG2 cells utilize micropinocytosis, and cardiomyocytes rely on actin-dependent internalization, Colo205 cells employ caveolae-dependent endocytosis, and mesenchymal stem cells use clathrin-mediated endocytosis [101-104]. Proteins on the outer mitochondrial membrane and surface molecules on recipient cells, such as heparan sulfate, are crucial in these processes, indicating that mitochondrial internalization is highly dependent on receptor-ligand interactions [101]. Interestingly, injured endothelial cells are unable to integrate naked mitochondria, although they can internalize them *via* TNTs [66]. Post-translational modification of mitochondrial proteins is crucial for their proper function, stability, and regulation in various cellular processes. O‐linked‐β‐N‐acetylglucosamine (O‐GlcNAcylation) of mitochondrial proteins has been reported to support extracellular mitochondrial function, and decreased O-GlcNAcylation in astrocytes impairs the neuroprotective effects of exogenous mitochondria, potentially leading to neuronal cell death [105].

Efforts to optimize pharmaceutical formulations have been made to enhance therapies based on intercellular naked mitochondria transport. For example, linking mitochondria to ligands of cell-surface asialoglycoprotein receptors (AsGRs) can target the complex to hepatocytes, facilitating receptor-mediated endocytosis [106]. Although this pathway does not exist in the nervous system, alternative ligands and receptors may be utilized. Intranasal microinjection of naked functional mitochondria has also been explored as a non-invasive route for mitochondrial transfer [107]. The mechanisms underlying mitochondrial release, internalization, and activity maintenance, as discussed, could be leveraged to regulate intercellular naked mitochondria transport. However, these mechanisms have primarily been studied in cancer cells and platelets, and their applicability to the nervous system remains uncertain.

## 4 POTENTIAL OF MITOCHONDRIAL TRANSPORT IN CEREBRAL ISCHEMIA TREATMENT

 Mitochondrial transfer is integral to various physiological processes and disease progression, interacting with endogenous MQC mechanisms to maintain mitochondrial quality and function. This process involves supplementing recipient cells with healthy mitochondria and degrading damaged mitochondria through classic pathways. As a result, targeting mitochondrial transfer has emerged as a promising therapeutic strategy. Current therapeutic approaches to mitochondrial transfer are relatively limited, primarily focusing on supplementing mitochondria and modulating their transfer mechanisms [108]. These strategies not only promote the recovery of mitochondrial dynamics and autophagy in recipient cells but also offer new insights and directions for the treatment of related diseases. Although intercellular mitochondrial transfer occurs infrequently in healthy brains, its significance becomes increasingly prominent under pathological conditions. To improve the effectiveness of mitochondrial transfer, researchers are actively exploring various methods, including pharmacological interventions and brain mitochondrial transplantation strategies (Table **2**).

### 4.1 Therapeutic Strategies Targeting Intercellular Mitochondria Transfer

Under normal physiological conditions, mitochondrial transfer between cells is a relatively stable and regulated process that facilitates cooperation and functional complementarity between cells. However, under stress, the rate of mitochondrial transfer increases significantly as part of an adaptive response to various stimuli. During these conditions, cells can enhance mitochondrial transfer and exchange by increasing the number of TNTs and EVs or by releasing naked mitochondria [78, 109-111]. As previously discussed, *in vitro* induction of TNT formation can be achieved by transfecting cells with proteins involved in cytoskeletal mobility and cell adhesion, such as CD38 [84], Wnt ligands [70], Eps8 [71], Cx43 [112], Arp2/3 activators [113], and Miro1 [114]. Additionally, various types of nanomaterials have been developed to promote TNT formation and thereby enhance mitochondrial transfer [115]. However, while nanomaterial-based approaches can effectively induce TNT formation, concerns regarding nanomaterial-induced neurotoxicity remain.

### 4.2 Mitochondrial Transplantation

Conversely, mitochondria transplantation appears to be more feasible. Local injection of astrocyte-derived mitochondria into the peri-infarct cortex has been shown to significantly upregulate cell survival-related signals, such as phosphorylated Akt and Bcl-xl, thereby promoting neuronal survival [83]. Transplanted mitochondria can restore mitochondrial function and alleviate oxidative stress in neurons, leading to a reduction in infarct size and blood-brain barrier leakage [116]. Mesenchymal stem cells, known for their high mitochondrial transfer capacity, favorable biosafety profile, and wide availability, are increasingly used as donor cells for mitochondrial transfer. Inhibition of mitochondrial function in mesenchymal stem cells has been shown to reduce infarct volume in ischemic mice, alleviate behavioral disorders, and reduce anxiety-like behaviors caused by stroke [117, 118]. Overexpression of VEGF further enhances the neuroprotective effect of mitochondrial transplantation using mesenchymal stem cells [117]. However, these methods are invasive, and due to the potential for tissue damage, their clinical translation remains limited. Less invasive methods, such as intravenous infusion [79] and intranasal administration [119], have also been reported to allow transplanted mitochondria to enter the brain and exert protective effects. Nevertheless, achieving specificity or targeted delivery of transplanted mitochondria within the brain remains challenging due to the uneven distribution and uptake of mitochondria, as well as the difficulty in targeting specific cell populations and brain regions affected by diseases. EVs serve as natural nanocarriers for drug delivery, exhibiting excellent biocompatibility *in vivo* and offering significant advantages in the treatment of ischemic stroke. By modifying their membranes, delivery efficiency can be greatly enhanced [120]. Furthermore, co-encapsulating therapeutic cargoes with mitochondria in EVs can substantially boost therapeutic efficacy [121].

## CONCLUSION

Mitochondria, the power plants of cells, are crucial regulators of various physiological processes, including apoptosis, oxidative stress, and calcium ion balance. To maintain their quantity and quality, mitochondria undergo dynamic changes such as fission and fusion. To maintain intracellular homeostasis, dysfunctional mitochondria undergo processes such as mitophagy or are actively released. In ischemic neurons, axonal mitochondria are transported back to the neuron’s soma for autophagy rather than being cleared at the axon site. Damaged mitochondria that are not transported back are degraded *via* transmitophagy, a process involving cells with high phagocytic activity, such as astrocytes and microglia within the nervous system. Following the internalization of dysfunctional mitochondria, mitochondrial biosynthesis in astrocytes is upregulated, potentially providing energy for the disposal of exogenous particles or serving as an intercellular supplement of functional mitochondria for adjacent injured cells. Conversely, if the endogenous lysosomal autophagy pathway fails to eliminate these damaged components, they can be released into the brain milieu, potentially exacerbating the pathological condition.

At the same time, mitochondria exchange between cells has been observed in both physiological and pathological conditions. When mitochondrial stress or energy shortages occur, intercellular mitochondrial transfer serves as a compensatory mechanism, allowing the transfer of healthy mitochondria from donor cells to recipient cells, thereby restoring mitochondrial function. This process is particularly significant in the context of brain injuries, such as hypoxia and ischemia, where healthy mitochondria are transported to damaged neurons to promote repair, regeneration, and functional recovery. Currently, functional mitochondria are known to be transferred *via* TNTs, EVs, or naked mitochondria. Reinforced mitochondria supplementation has been considered a potential strategy for treating neurological disorders. From an evolutionary perspective, species adapted to chronic hypoxia exhibit unique mitochondrial adaptations. For instance, the brain mitochondria of red-eared slider turtles exhibit reduced citrate synthase and F1F0-ATPase activity, along with mild proton gradient decoupling [122]. Animals experiencing low-oxygen environments during development, such as snapping turtles, demonstrate lower leakage respiration, higher P:O ratios, and reduced ROS production rates in adulthood [123]. Additionally, flies adapted to hypoxic environments have been shown to reduce electron transport chain complex activity, mitochondrial metabolism, and ROS production [124]. Mitochondrial transfer may also be involved in mitochondrial adaptation in chronically hypoxic environments.

Mitochondrial transfer plays a vital role in MQC. While research is advancing our understanding of the mechanisms underlying mitochondrial transfer and potential therapeutic strategies, several critical questions remain unanswered before clinical translation can be realized. It remains unclear whether mitophagy through transport back to the soma and mitochondrial retention in the axon coexist or whether they play dominant roles in different pathological processes. Additionally, the initiation signals and regulatory factors of mitochondrial transfer between different cells have not been fully elucidated. Although it is known that mitochondrial transfer is influenced by cellular metabolic needs, stress states, and other factors, the specific signals that trigger mitochondrial transfer, as well as how these signals are perceived and transmitted, remain unclear.

Furthermore, the fate of transferred mitochondria is not well understood. Whether these mitochondria integrate into the reci484-493pient cell's mitochondrial network, are degraded and cleared, or assume other functions remains to be determined. The long-term effects of mitochondrial transfer on recipient cells, including its impacts on cell metabolism, signaling, and gene expression, also warrant further exploration.

Mitochondrial transfer therapy for cerebral ischemia has shown promising initial results in animal models. However, challenges such as low delivery efficiency and targeting specificity persist, necessitating further clinical studies to accelerate the development of mitochondrial transfer therapies.

## Figures and Tables

**Table 1:** The intercellular supplement of functional mitochondria in stroke.

**Type of Transport**	**Donor Cell**	**Recipient Cell**	**Models**	**Methods**	**References**
TNTs	Human neural stem cells	Human neuroblastoma SHSY5Y cells	Oxygen-glucose deprivation/ Reoxygenation	Direct contact co-culture & Non-contact co-culture	[63]
	Cath.-a-differentiated cells	Cath.-a-differentiated cells	H_2_O_2_ treatment	Direct contact co-culture	[55]
	Bone marrow mesenchymal stem cells	-	Middle cerebral artery occlusion	BMSCs transplantation	[117]
	Multipotent mesenchymal stem cells	Neuron-like PC12 cells & primary astrocyte	Oxygen-glucose deprivation	Direct contact co-culture	[60]
		-	Middle cerebral artery occlusion	MMSCs injection (i.v.)	
	Human marrow stromal cells	Primary neuron	H_2_O_2_ treatment	Direct contact co-culture & Non-contact co-culture	[125]
**-**	Primary astrocyte	Primary neuron	Oxygen-glucose deprivation/ Reoxygenation	Direct contact co-culture	[83]
	HT22 cells	HT22 cells	Oxygen-glucose deprivation/ Reoxygenation	Direct contact co-culture	[126]
	Primary astrocyte	Primary neuron	Oxygen-glucose deprivation/ Reoxygenation	Direct contact co-culture	[62]
	Human cerebral microvascular endothelial cells	Normal human brain astrocytes	Oxygen-glucose deprivation	Direct contact co-culture	[127]
	Human astroglioma U251 cells	Human neuroblastoma SHSY5Y cells	CoNPs treatment	Direct contact co-culture	[65]
	Human umbilical vein endothelial cells	Human mesenchymal stem cells	Oxygen-glucose deprivation/ Reoxygenation	Direct contact co-culture	[66]
	Primary astrocytes	Primary neurons	H_2_O_2_ treatment	Direct contact co-culture	[67]
	U-87 MG cells	U-87 MG cells	H_2_O_2_ treatment	Direct contact co-culture	[128]
EVs	Primary astrocyte	Primary neuron	Oxygen-glucose deprivation/ Reoxygenation	Astrocyte-conditioned media culture	[83]
	Blood-brain barrier (hCMEC/D3) cells	Human cerebral microvascular endothelial cells & Blood-brain barrier (hCMEC/D3) cells	Middle cerebral artery occlusion	Isolated mito-EVs injection (i.v.)	[79]
			Oxygen-glucose deprivation/ Reoxygenation	Isolated mito-Evs incubation	
	Human placenta stem cells	R28 retinal precursor cells	Hypoxia treatment	Isolated mito-Evs incubation	[129]
	Mesenchymal stem cells	Primary hippocampal neuron	H_2_O_2_ treatment	Isolated mito-Evs incubation	[81]
Naked mitochondria transport	Human endothelial progenitor cells	Brain endothelial cells	Oxygen-glucose deprivation/ Reoxygenation	EPCs-conditioned media culture	[95]
	Mitochondria isolated from mice cortex	Primary neuron	Oxygen-glucose deprivation/ Reoxygenation	Isolated mitochondria incubation	[130]
		-	Middle cerebral artery occlusion	Isolated mitochondria injection (i.v.)	
	Primary astrocyte	Primary neuron	Oxygen-glucose deprivation/ Reoxygenation	Isolated astrocytic mitochondria incubation	[105]
	Xenomitochondria from hamster cells	Neuron and astrocyte	Middle cerebral artery occlusion	Isolated mitochondria injection (i.v.)	[97]

**Table 2:** Therapeutic strategies targeting mitochondria transfer in stroke.

**Therapeutic Strategies**	**Source of Mitochondria**	**Inventions**	**Outcome**	**References**
Pharmacologic Approaches	Primary astrocyte	CD38-cADPR-calcium signaling activation	Increased astrocyte-neuron mitochondria transfer, promoted neuron survival & dendrite regrowth	[84]
	Primary astrocyte	Mitochondrial protein O-GlcNAcylation	Increased astrocyte-neuron mitochondria transfer & promoted neuron survival	[105]
	Mesenchymal stem cells	Miro1 overexpression	Increased mitochondrial transfer & improved survival of cisplatin-treated NSCs	[114]
	Human bone marrow MMSC	Miro1 overexpression	Promoted MMSC-astrocyte mitochondrial transfer & improved recovery of neurological functions	[60]
	Primary neuron	GJA1-20K/Cx43 axis activation	Promoted astrocyte-astrocyte mitochondrial transfer & promoted neuron survival	[112]
	Primary microglia	Mitochondria fission inhibition	Inhibited dysfunctional mitochondria release, inflammation & reduced neuronal cell death	[45]
Invasive Mitochondria Transplantation	Primary mouse astrocytes	Local injection into peri-infarct cortex	Upregulation of cell survival-related signals	[83]
	Bone marrow mesenchymal stromal cells	Local injection into peri-infarct striatum	Attenuated behavioral defects & reduced infarct volume 7 days after stroke	[117]
	Isolated mitochondria from the liver and muscle	Local injection into peri-infarct cortex	Alleviate trauma-associated anxiety, improve spatial memory & cognitive function	[118]
	Isolated mitochondria from mesenchymal stem cells	Local injection into the right lateral ventricle	Decreased astrogliosis, microglia, activation, reduced infarct size & improved motor function	[131]
	Isolated mitochondria from hamster kidney fibroblast	Local injection into peri-infarct cortex & intra-arterial injection	Restored the motor performance, attenuated brain infarct area & neuronal cell death	[97]
	Isolated mitochondria from pectoralis	Local injection into peri-infarct cortex	Reduced cellular oxidative stress, apoptosis & attenuated reactive astrogliosis	[132]
	Brain-derived mitochondria	Local injection into peri-infarct cortex	Reduced cellular apoptosis, promoted angiogenesis, alleviated brain edema & blood-brain barrier leakage	[116]
Noninvasive Mitochondria Transplantation	Isolated mitochondria from human brain endothelial cells	Injection after reperfusion (i.v.)	Reduced brain infarct sizes	[79]
	Isolated mitochondria from multipotent mesenchymal stem cells	Injection after reperfusion (i.v.)	Reduced dysfunction of the limb innervated from the damaged regions of the brain cortex	[60]
	Isolated mitochondria from cryopreserved mouse placenta	Injection after reperfusion (i.v.)	Decreased infarction after focal cerebral ischemia in mice	[133]
	Isolated mitochondria from primary astrocyte	Injection (i.v.)	Restored Mn-SOD levels & improved functional recovery	[134]
Extracellular Carrier Modification	Isolated mitochondria from intact cerebral cortex with DOTAP coat	Injection after reperfusion (i.v.)	Amplified cerebral protection *in vivo*	[130]
	Mitochondria loaded with mixtures of EV/exogenous HSP27	Injection after reperfusion (i.v.)	Reduced brain infarct sizes	[79]

## LIST OF ABBREVIATIONS

AD: Alzheimer's Disease
ALS: Amyotrophic Lateral Sclerosis
DANs: Dopaminergic Neurons
MQC: Mitochondrial Quality Control
PD: Parkinson's Disease
